# A High-Performance Multiplex Immunoassay for Serodiagnosis of Flavivirus-Associated Neurological Diseases in Horses

**DOI:** 10.1155/2015/678084

**Published:** 2015-09-17

**Authors:** Cécile Beck, Philippe Desprès, Sylvie Paulous, Jessica Vanhomwegen, Steeve Lowenski, Norbert Nowotny, Benoit Durand, Annabelle Garnier, Sandra Blaise-Boisseau, Edouard Guitton, Takashi Yamanaka, Stéphan Zientara, Sylvie Lecollinet

**Affiliations:** ^1^UMR 1161 of Virology, ANSES, INRA, ENVA, ANSES Animal Health Laboratory, EU-RL on Equine Diseases, UPE, 94701 Maisons-Alfort, France; ^2^UMR PIMIT (I2T Team), INSERM U1187, CNRS 9192, IRD 249, Technology Platform CYROI, University of Reunion, 97491 Saint-Clotilde, Réunion; ^3^Department of Infections and Epidemiology, Institut Pasteur, 75724 Paris, France; ^4^Viral Zoonoses, Emerging and Vector-Borne Infections Group, Institute of Virology, University of Veterinary Medicine Vienna, 1210 Vienna, Austria; ^5^Department of Microbiology and Immunology, College of Medicine and Health Sciences, Sultan Qaboos University, 123 Al-Khoudh, Oman; ^6^INRA UE 1277, Plate-Forme d'Infectiologie Expérimentale, 37380 Nouzilly, France; ^7^Equine Research Institute, Japan Racing Association, Tochigi 329-0412, Japan

## Abstract

West Nile virus (WNV), Japanese encephalitis virus (JEV), and tick-borne encephalitis virus (TBEV) are flaviviruses responsible for severe neuroinvasive infections in humans and horses. The confirmation of flavivirus infections is mostly based on rapid serological tests such as enzyme-linked immunosorbent assays (ELISAs). These tests suffer from poor specificity, mainly due to antigenic cross-reactivity among flavivirus members. Robust diagnosis therefore needs to be validated through virus neutralisation tests (VNTs) which are time-consuming and require BSL3 facilities. The flavivirus envelope (E) glycoprotein ectodomain is composed of three domains (D) named DI, DII, and DIII, with EDIII containing virus-specific epitopes. In order to improve the serological differentiation of flavivirus infections, the recombinant soluble ectodomain of WNV E (WNV.sE) and EDIIIs (rEDIIIs) of WNV, JEV, and TBEV were synthesised using the *Drosophila* S2 expression system. Purified antigens were covalently bonded to fluorescent beads. The microspheres coupled to WNV.sE or rEDIIIs were assayed with about 300 equine immune sera from natural and experimental flavivirus infections and 172 nonimmune equine sera as negative controls. rEDIII-coupled microspheres captured specific antibodies against WNV, TBEV, or JEV in positive horse sera. This innovative multiplex immunoassay is a powerful alternative to ELISAs and VNTs for veterinary diagnosis of flavivirus-related diseases.

## 1. Introduction

Many flaviviruses such as West Nile virus (WNV), tick-borne encephalitis virus (TBEV), Japanese encephalitis virus (JEV), or dengue virus are emerging or reemerging diseases threatening humans and/or animals [1–4]. Most flaviviruses are arboviruses that can be transmitted by ticks,* Aedes* or* Culex* mosquitoes, or by unknown vectors [5, 6]. Mosquito-borne viruses are grouped into serocomplexes such as the dengue or Japanese encephalitis serocomplexes on the basis of their antigenic relationships [7]. WNV and JEV—two major encephalitic viruses in horses and humans [8, 9]—belong to the Japanese encephalitis serocomplex which is composed of eight virus species and two subtypes [10]. TBEV, another encephalitic flavivirus known to cause severe infections in humans and dogs, belongs to the group of mammalian tick-borne viruses [2].

Only low levels of viremia are induced after infection with most flaviviruses (apart from dengue), so diagnosis is confirmed mainly through serological tools. Rapid serological tools are widely used in human and veterinarian diagnostic laboratories, in particular enzyme-linked immunosorbent assays (ELISAs) and immunofluorescence assays (IFAs). ELISAs and IFAs are very sensitive but suffer from a lack of specificity, generating positive reactions for close flaviviruses within a specific serocomplex or even for flaviviruses belonging to other serocomplexes [6]. For example, JEV- or TBEV-infected patients can generate false positive reactions in WNV ELISAs [11, 12]. Serological assays should thus be interpreted with care taking into account the patient's history of vaccination and past infection and confirmed by comparative virus neutralisation tests (VNTs) [7, 13]. Nevertheless, the VNT is usually less sensitive than ELISA or IFA and some residual cross-reactions can be observed within the Japanese encephalitis serocomplex. This time-consuming immunoassay generally requires the production of infectious virus particles in BSL3 facilities.

Rapid flavivirus serological assays have already been misinterpreted in the past, highlighting the need for improved serological tools. During the emergence of WNV in the USA in 1999, the first WNV cases were initially thought to be Saint Louis encephalitis cases, a flavivirus prevalent in North America [14]. Consequently, the flavivirus surveillance programmes currently in operation in many countries could detect belatedly, or even fail to detect, the emergence of a new flavivirus. Moreover, the spatial and temporal overlapping in the circulation of flaviviruses makes it difficult to precisely monitor the dynamics of a specific flavivirus. Mosquito-borne flaviviruses like WNV or Usutu virus are widely distributed throughout Europe with high spatial overlapping, for example, in Italy, Austria, and Hungary [15–17], while WNV and TBEV are regularly isolated in Central Europe [6].

Most antibodies elicited during flavivirus infections are directed against the highly immunogenic envelope (E) protein, which contains both flavivirus cross-reactive and virus-specific epitopes [18, 19]. The E glycoprotein is composed of three domains named EDI, EDII, and EDIII, altogether composing the soluble ectodomain of E (sE). Both EDI and EDIII contain virus-specific epitopes [20–22], and EDIII mediates virus attachment to the cell membrane [23, 24]. Potent neutralising antibodies have been shown to map to EDIII [25–29], and amino acid substitutions within EDIII may influence the pathogenicity of flaviviruses [27, 30–34].

The development of xMAP microsphere immunoassays (MIAs) or Luminex assays for human and veterinary diagnoses offers promising multiplexing approaches to the capture of specific antibodies directed against flavivirus antigens [35–38]. To significantly improve the specificity and sensitivity of flavivirus serodiagnosis based on the capture of specific antibodies in host vertebrates, MIA has been designed with recombinant flavivirus EDIII antigens (rEDIII) covalently coupled to colour-coded microspheres. This innovative diagnostic tool combines the ease and rapidity of ELISA with the precision of flow cytometry technology, giving a reliable measurement of the multiple components of antibody response in a single assay.

The serological diagnosis of infection due to neurotropic flaviviruses is challenging due to their antigenic cross-reactivity [11, 12, 39, 40]. The rapid serological tests used to diagnose tick-borne encephalitis in Europe may be confused with West Nile disease in endemic regions with high prevalence rates of infection in humans and horses [2, 41–43]. This research evaluated the capacity of the rEDIII-based MIA to detect and differentiate the antibody response induced after three flavivirus infections responsible for encephalitis in humans and horses: WNV, JEV, and TBEV.

Equine reference sera for these three diseases were produced by experimental infection. These experiment-produced sera were tested with an innovative MIA, as were field sera collected from horses in countries with highly prevalent JEV (Japan), TBEV (Austria), or WNV (Madagascar and Pakistan) infections. The results obtained were compared using VNTs for WNV, JEV, and TBEV and a flavivirus competition ELISA.

## 2. Materials and Methods

### 2.1. Production of Recombinant WNV.sE and rEDIIIs of WNV, JEV, and TBEV

The* Drosophila* S2 expression system (DES, Life Technologies) was used to produce recombinant WNV.sE and the flavivirus rEDIIIs in* Drosophila *S2 cells.

The synthetic genes (GeneCust, Luxembourg) encoding the soluble ectodomain of E from WNV strain IS-98-ST1 (Genbank AY033389.1, residues E-1 to E-405) and the EDIII (residues E-295 to E-405) from IS-98-ST1, EDIII (residues E-302 to E-413) from JEV strain GP05 of genotype 3 (Genbank FJ979830.1), or EDIII (residues E-293 to E-399) from TBEV strain Kumlinge A52 (Genbank AY268437.1) were cloned into shuttle vector pMT/BiP/HisA in which the SNAP-tag sequence (Covalys BioSciences AG) had been initially inserted as a stabilizing protein. The resulting plasmids encoding either chimeric protein WNV.sE-SNAP or chimeric proteins SNAP-EDIII were transfected into S2 cells to establish stable cell lines S2/WNV.sE, S2/WNV.EDIII, S2/JEV.EDIII, and S2/TBEV.EDIII according to the manufacturer's recommendations (Life Technologies) [44]. After a 10-day cadmium induction of the stable S2 cell lines grown in a 1 L spinner, cell supernatants were recovered and secreted soluble His-tagged recombinant viral antigens were purified on chelating and size-exclusion chromatography columns. The protein quantities of highly purified WNV.sE-SNAP, SNAP-WNV.EDIII, SNAP-JEV.EDIII, and SNAP-TBEV.EDIII were determined using a BCA protein assay kit (Thermo Scientific) according to the manufacturer's instructions. Ten to hundred milligrams of recombinant viral antigens was obtained from a single 1 L batch of S2 cell culture (Table 1).

### 2.2. Western Blot

**Figure 1:**
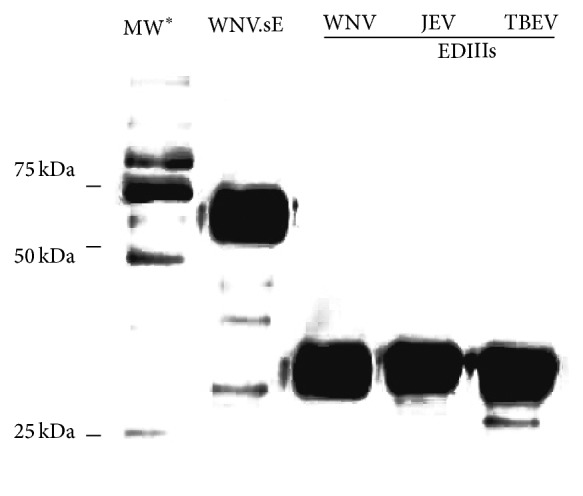
12% polyacrylamide gel electrophoresis showing the recombinant WNV.sE-SNAP, SNAP-WNV.EDIII, SNAP-JEV.EDIII, and SNAP-TBEV.EDIII proteins after purification. ^*∗*^MW: molecular weight marker.

A Western blot analysis was carried out on 12% polyacrylamide gel on purified proteins heated for five minutes at 95°C and mixed with Laemmli 1X (Bio-Rad) plus 5% *β*-mercaptoethanol. Proteins were subsequently transferred to nitrocellulose membrane and probed overnight at 4°C with the anti-His mouse monoclonal antibody at a dilution of 1 : 1000 (Invitrogen, Life Technologies). The membranes were incubated at a 1 : 5000 dilution of anti-rabbit immunoglobulins coupled to horseradish peroxidase (DakoCytomation) for one hour at room temperature (RT). Proteins were then detected with Clarity ECL Western blotting substrate (Bio-Rad) with five minutes of incubation and 30 seconds of exposure. The Precision Plus Protein WesternC Standard was used as a molecular size marker (Bio-Rad) (Figure 1).

### 2.3. Multiplex Immunoassay Technology

#### 2.3.1. Coupling Assay

Different colour-coded magnetic beads (Bio-Plex ProTM Magnetic COOH Beads, Bio-Rad Laboratories) were coupled at room temperature (RT) to WNV.sE or flavivirus rEDIIIs through carboxylate amine bonds. Bead regions 34, 46, 53, and 65 were used for TBEV.EDIII, JEV.EDIII, WNV.sE, and WNV.EDIII, respectively. The amine coupling assay was performed following the manufacturer's instruction manual using the commercial Bio-Plex Amine Coupling Kit (Bio-Rad Laboratories).

Each coupling reaction required 1.25 × 10^6^ beads. Beads were vortexed thoroughly for one minute and washed twice with the bead wash buffer using a magnetic separator to retain the beads. The beads were then resuspended in 80 *μ*L of bead activation buffer. Ten microlitres of hydroxysulfosuccinimide sodium salt (S-NHS; 50 mg/mL) (Sigma-Aldrich) and 1-ethyl-3-(3-dimethylaminopropyl)carbodiimide hydrochloride (EDAC; 50 mg/mL) (Sigma-Aldrich) was prepared extemporaneously in the bead activation buffer and added to the bead suspensions. The beads were vortexed and then placed in the dark on a rotary wheel for 20 min at RT. One hundred and fifty microlitres of PBS at pH 7.4 (Gibco, Life Technologies) was added to the bead, briefly mixed by vortexing at high speed, and removed (step repeated once). The beads were then resuspended in 100 *μ*L of PBS. Five micrograms of WNV.sE or TBEV.EDIII or 50 *μ*g of WNV.EDIII or JEV.EDIII (optimal final protein amount established after different assays) was added to the beads in 500 *μ*L of PBS (pH 7.4). The beads were incubated in the dark on a rotary wheel for 2 h. The coupled beads were washed with 500 *μ*L of PBS (pH 7.4), resuspended in 250 *μ*L of blocking buffer, vortexed, and then incubated for 30 min in the dark on a rotary wheel. The supernatant was removed and the coupled microspheres were resuspended in 500 *μ*L of storage buffer, vortexed, removed, and finally resuspended in 150 *μ*L of storage buffer, counted, and stored between 2 and 8°C in the dark.

#### 2.3.2. Multiplex Immunoassay with WNV.sE and Flavivirus rEDIIIs

**Figure 2:**
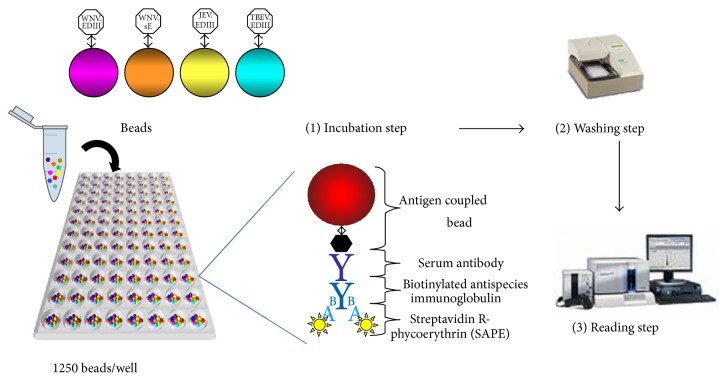
Presentation of the flavivirus microsphere immunoassay (MIA) with four beads coupled to four antigens (WNV.sE, WNV.EDIII, JEV.EDIII, and TBEV.EDIII).

The magnetic beads coupled to their specific antigen were diluted in 50 *μ*L of dilution buffer from the ID Screen West Nile competition kit (ID Vet Company) at a final concentration of 1250 beads/well and placed in the wells of the MIA plate (Bio-Plex ProTM flat bottom well plates, Bio-Rad Laboratories). Serum samples and controls, once heat-inactivated for 30 min at 56°C ± 1°C, were diluted at 1 : 100 in 50 *μ*L of the ID Screen dilution buffer and mixed with the beads. After one-hour incubation at RT on a plate shaker, the plate was washed three times with the washing solution (PBS containing 0.05% Tween-20). Fifty microlitres of a secondary biotinylated goat anti-horse IgG (Jackson Immuno Research Inc.), diluted at 1 : 500 in the ID Screen dilution buffer, was added to each well and incubated at RT for 45 min on a plate shaker. After three washing steps, 50 *μ*L of streptavidin R-phycoerythrin conjugate (SAPE; 1 *μ*g/mL; Qiagen), diluted at 1 : 100 in the ID Screen dilution buffer, was added to each well. Finally, after 15-minute incubation at RT on a plate shaker and three additional washing steps, the beads were resuspended in 125 *μ*L of xMAP Sheath fluid buffer (Bio-Rad Laboratories) and analysed using a Luminex 200 system (Bio-Rad Laboratories) (Figure 2). Fluorescence intensity and bead colour coding were measured by dual lasers that excite the beads at two different wavelengths: 635 nm to identify the microsphere particle and 532 nm to excite the SAPE reporter dye [45].

For each sample, the median fluorescence intensity (MFI) was calculated from at least 50 beads bearing the same antigen.

All equine sera were tested in duplicate in two separate assays. If discrepant results were obtained from the two assays, the corresponding sample was retested. The results presented below correspond to one representative MIA.

### 2.4. ELISA Procedure

Equine serum samples were screened using a commercial ELISA (ID Screen West Nile competition ELISA kit; ID Vet, Montpellier, France) for antibodies against WNV and related flaviviruses [11, 13, 40, 41, 46, 47]. Assays were performed according to the manufacturer's instructions. In particular, the threshold value for considering a serum as positive by the competition ELISA was % S/N < 40% and as doubtful was 40 ≤ % S/N < 50% as recommended by the manufacturer.

### 2.5. Virus Neutralisation Tests

ELISA positive samples were further investigated through virus-specific microneutralisation tests (MNTs) against flaviviruses reported in the area where the sera were collected.

Heat-inactivated sera, serially diluted (1 : 5 to 1 : 3645) in Dulbecco's modified Eagle's medium (DMEM) were mixed with an equal volume (50 *μ*L) of DMEM containing 100 tissue culture infectious dose 50 (TCID50) of WNV strain IS-98-ST1, JEV strain Nakayama, Usutu virus (USUV) strain SAAR-1776 (South Africa) or TBEV strain Hypr (most being a generous gift from the National Reference Centre for Arboviruses in France at the Institut Pasteur, Paris). Each serum was tested in duplicate. Cell and virus controls, as well as virus back titration controls, were included in every MNT run. After incubation of the plates at 37°C for 1.5 h, 2 × 10^4^ Vero cells in 100 *μ*L of DMEM were added to all the wells. Plates were incubated at 37°C for 3 to 5 days (3 days for WNV and USUV, 4 days for JEV and 5 days for TBEV), then cytopathogenic effects were observed under a light microscope. A serum was considered negative if infection occurred regardless serum concentration. It was considered positive if cells were protected at the 1 : 10 serum dilution for WNV, USUV and JEV and 1 : 20 for TBEV; its titre was calculated as the inverse of the latest dilution at which cells were protected [48]. For TBEV field sera from Austria, TBEV antibodies were previously identified by a Plaque Reduction Neutralisation Test (PRNT) as per an existing protocol [41].

Owing to cross-neutralisation between flaviviruses, especially within the same serocomplex, VNTs end point titres need to be compared. The flavivirus can then be identified by considering the virus with the highest neutralisation capacity and at least a fourfold difference in titres [49, 50].

### 2.6. Serum Samples

#### 2.6.1. Reference Sera

Four Welsh mares of varying ages (between 7 and 17 years) were used for experimental infection with different flaviviruses. The ponies were screened for the presence of antibodies against WNV or related flaviviruses using a commercial ELISA kit (ID Screen West Nile competition ELISA kit; ID Vet) and found negative prior to flavivirus infection. One week before infection, they were moved into a biocontainment building at the experimental platform of infectiology (INRA, Nouzilly) and maintained under biosafety level 3 conditions for 10 weeks. They were infected subcutaneously with 10^7^ pfu of either WNV lineage 1 (WNV1, IS-98-ST1 strain), or lineage 2 (WNV2, Aus08), or JEV (Nakayama strain) or TBEV (Hypr strain). The animals were monitored for clinical signs for 10 weeks and blood was sampled on day 0 (D0), day 4 (D4), day 8 (D8), day 11 (D11), day 14 (D14), day 20 (D20), day 35 (D35), and day 58 (D58). Sera were obtained by centrifuging blood samples (2400 g, 10 minutes) and tested with the ID Screen West Nile competition kit (ID Vet), MIA, and MNT (viruses tested: WNV, JEV, and TBEV). These infections were initiated after bioethics acceptance according to European regulations on animal welfare (Directive 2010/63/UE; French Ethical Committee number 23-01-6V2).

#### 2.6.2. Field Sera

A panel of WNV, JEV, and TBEV positive and negative field sera were tested with the developed MIA.

*WNV Sera*. One hundred and one field sera collected in either Madagascar (35) or Pakistan (66) were found positive with the ID Screen West Nile competition kit (ID Vet), and two sera were doubtful. Prior to MIA testing, sera were evaluated by MNTs to investigate the causative flavivirus involved. According to the known circulation history of flaviviruses in each area, WNV and USUV MNTs were performed on the sera from Madagascar, while WNV and JEV antibodies were evaluated in the sera from Pakistan. JEV neutralizing antibodies were also sought in the sera from Madagascar due to the substantial propensity of JEV to spread. WNV infection was confirmed by MNT on 96 Equidae from Pakistan [51] and Madagascar.

*JEV Sera*. One hundred and one field sera were sampled in horses injected twice at one-month intervals with the inactivated Japanese encephalitis vaccine (Nisseiken, Tokyo) according to the manufacturer's instructions, about four months after the last vaccination, and were sent by the Japan Racing Association. They were tested simultaneously by ELISA, MNT (viruses tested: JEV and WNV), and flavivirus MIA. JEV-specific antibodies were identified in 91 sera by the MNT. 

*TBEV Sera*. Sera were collected from 74 Lipizzaner horses spread over three federal states in Austria and then screened using the ID Screen West Nile competition kit (ID Vet). ELISA positive and negative serum samples were further investigated by a VNT for the three flaviviruses circulating in Austria (WNV, USUV (MNT), and TBEV (PRNT)). TBEV-specific antibodies were identified in 62 sera by PRNT [41].

*Negative Sera*. To test the specificity of the method, 104 field sera sampled in the Camargue region of Southern France in 2007 and 56 sera from Ireland—all found negative with the ID Screen West Nile competition kit (ID Vet)—were tested with the flavivirus MIA.

## 3. Results and Discussion

### 3.1. Coupling of Recombinant Viral Antigens on Coloured Microspheres

To significantly improve the specificity and sensitivity of flavivirus serodiagnosis based on the capture of specific antibodies in host vertebrates, the MIA was designed with recombinant flavivirus EDIII antigens (rEDIII) covalently coupled to colour-coded microspheres. Amino acid similarities in the EDIII proteins of JEV, WNV, and TBEV were determined using NCBI, Blastp program. Sequence alignment showed an amino acid identity of 39% for WNV and JEV versus TBEV and 74% between WNV and JEV, as previously determined by Danecek et al. [30]. Highly concentrated recombinant WNV.sE and rEDIII proteins from JEV, WNV, and TBEV were obtained in the supernatant of S2 cells by using the* Drosophila* S2 expression system (Table 1) and were purified on chromatography columns. The apparent molecular weights (MWs) of the soluble WNV.sE-SNAP protein (between 50 and 75 kDa) and SNAP.EDIII proteins (between 25 and 37 kDa) on PAGE-SDS were consistent with their theoretical MWs (Figure 1).

### 3.2. Determination of MIA Experimental Cut-Offs

The diagnostic cut-off of the flavivirus MIA technology was determined by ROC analysis for all the antigens using well-characterised sera found positive or negative by ELISA, for example, 172 negative samples from Ireland, Camargue (France), and Austria, 101 ELISA positive samples from Madagascar and Pakistan (for WNV.sE and WNV.EDIII cut-off), 88 positive samples from Japan (for WNV.sE and JEV.EDIII cut-off), and 59 positive sera from Austria (for TBEV.EDIII cut-off) (Table 2).

One low positive control for each bead was systematically included in every test and the fluorescence results obtained with WNV.sE, WNV.EDIII, JEV.EDIII, and TBEV.EDIII beads were normalised as the ratio of the sample MFI over the positive control MFI ×100 (S/P ratio).

**Figure 3:**
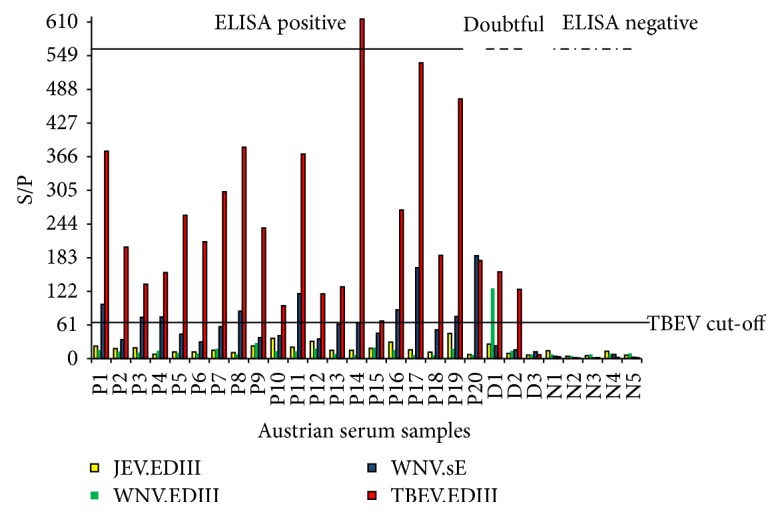
MIA fluorescence results, expressed as the ratio of the sample MFI over the positive control MFI X 100 (S/P ratio) on 20 ELISA positive (P), three doubtful (D), and five negative (N) horse samples from Austria, as determined with the ID Screen West Nile competition ELISA kit (ID Vet). The cut-off for the TBEV.EDIII antigen was determined to be 61.

The cut-off for the TBEV.EDIII antigen was determined to be 61, generating a diagnostic sensitivity (Se) of 98.4% and a specificity (Sp) of 100% (Figure 3). The cut-off for the WNV.sE antigen was determined to be 17, with corresponding Se of 98.4% and Sp of 99.4%, while cut-off values for WNV.EDIII and JEV.EDIII antigens were found to be 54 and 55, respectively, with corresponding Se of 97.0 and 93.2% and corresponding Sp of 92.3 and 94.7% (Table 2).

According to ROC analysis, sera positive for flaviviruses belonging to the JEV serocomplex were detected with a higher specificity when WNV.sE beads were used rather than WNV.EDIII or JEV.EDIII beads. Consequently, to improve the specificity of the developed MIA for the diagnosis of flaviviruses in the JEV serocomplex, a decision algorithm was set up as follows: (1) an immune serum was considered to be positive for WNV or JEV antibodies only if it reacted with the WNV.sE antigen; (2) on the contrary an immune serum that did not recognize WNV.sE antigen was evaluated as a naive serum for both anti-WNV and JEV antibodies. Based on this algorithm, the impact of the lower specificity of WNV.EDIII (Sp = 92.3%) and JEV.EDIII (Sp = 94.7%) tests was reduced by the fact that false reactives with WNV.EDIII or JEV.EDIII were discarded if the WNV.sE signal was found to be negative.

Since TBEV belongs to another serocomplex, and the TBEV.EDIII test was highly specific (Sp = 100%), a serum was considered positive for TBEV when the TBEV.EDIII MFI value was above the threshold.

Finally, taking into account possible cross-reactions between rEDIIIs due to partial rEDIII homology, a horse was considered infected with a specific flavivirus if the corresponding bead coupled to rEDIII generated an S/P ratio at least 1.5-fold greater than that generated with the other rEDIII beads. If a 1.5-fold difference could not be achieved, the sample was considered to be infected with an undetermined flavivirus. The sample was also considered positive for an undetermined flavivirus if it reacted with WNV.sE but not with any of the rEDIIIs.

### 3.3. Evaluation of the MIA on Experimental Sera

Sera sampled from experimentally infected horses (WNV1, WNV2, JEV, and TBEV) were analysed by flavivirus MIA and compared to flavivirus competition ELISA and WNV, JEV, and TBEV MNTs.

**Figure 4:**
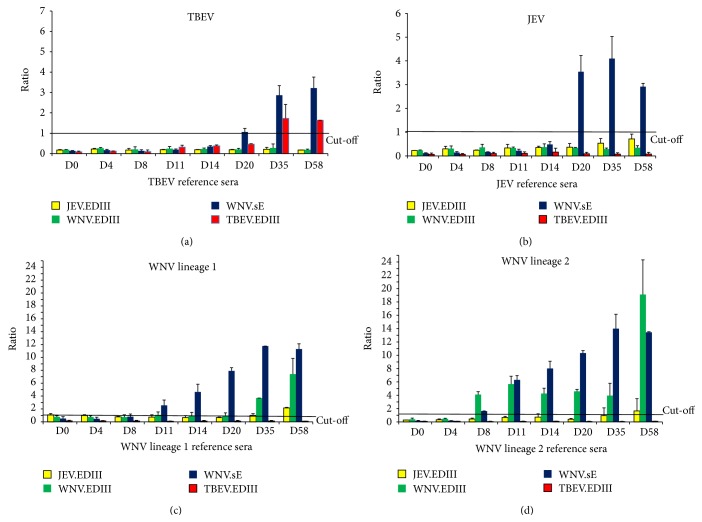
Reference equine sera sampled from ponies experimentally infected by different flaviviruses: TBEV (a), JEV (b), WNV lineage 1 (c), and WNV lineage 2 (d) collected on different days after infection and tested by flavivirus MIA with four antigen coupled beads (JEV.EDIII, WNV.EDIII, WNV.sE, and TBEV.EDIII). The mean and standard error of the S/P ratio of Ag “*x*” divided by the ROC cut-off value for Ag “*x*” is displayed. A sample was considered positive for the bead if its ratio was greater than one. The assay was carried out twice in separate experiments.

*Flavivirus Detection*. Given that the envelope E glycoprotein from WNV reacts to the antibodies directed at least against members of the JEV serocomplex of flaviviruses, the microspheres bearing the recombinant WNV.sE were more efficient in capturing early anti-WNV (lineage 1 and, to a lesser extent, lineage 2), anti-JEV, or anti-TBEV antibodies from infected ponies than the respective rEDIII beads (Figure 4). Antibody detection in WNV-infected ponies could be evidenced as early as 8–11 days postinfection (PI), which was comparable to MNT (D8) and ELISA (D11) results. For JEV infection, the three serological assays, that is, WNV.sE MIA, ELISA, and MNT, achieved a comparable detection efficacy as early as 20 days PI (Table 3). In TBEV-infected animals, anti-TBEV antibodies were more efficiently evidenced by a TBEV MNT (D8) than by ELISA or WNV.sE MIA detection (D20). TBEV belongs to the mammalian tick-borne encephalitis group and the percentage of homology between TBEV and WNV.sE proteins is lower than 40% (calculated by NCBI, Blastp program) which can account for the delay in detecting anti-TBEV antibodies by WNV.sE ELISA and MIA. To improve the early detection of TBEV, a TBEV.sE bead could be added to the panel of beads.

Therefore, in a single assay, the WNV.sE-based MIA was found to be more sensitive than ELISA for the detection of WNV lineage 2 antibodies and as sensitive as ELISA for the early detection of WNV lineage 1, JEV, and TBEV antibodies.

*Flavivirus Identification*. As mentioned above, a TBEV.EDIII positive signal alone is sufficient to consider a horse infected with TBEV, while WNV.EDIII and JEV.EDIII positive signals can be considered to be due to WNV or JEV infections if the sera also reacted with the WNV.sE coupled microspheres.

WNV antibodies were specifically identified as early as D8 PI in the pony infected with WNV lineage 2, which meant earlier detection than with the WNV MNT (D14). Conversely, TBEV-, JEV- and WNV-specific antibodies, in the case of the pony infected with a lineage 1 WNV strain, were identified later by MIA than by MNT (D8 for MNT versus D35 for MIA considering TBEV- and WNV lineage 1-infected ponies and D35 for JEV identification with MNT versus no identification with MIA) (Figure 4 and Table 3).

This discrepancy in early specific detection by MNT and MIA could arise from differences in the rapidity and intensity of antibody response in the immunised ponies. Indeed, only the pony having received the lineage 2 WNV developed signs of infection, with an increase in body temperature of 2°C nine days after the injection (data not shown). The fact that the JEV Nakayama strain used for JEV infection was an attenuated vaccine strain can also account for poor sensitivity in identifying JEV antibodies with the JEV.EDIII bead. Finally, EDIII IgM antibodies detected soon after infection in model or naturally infected mammals [18] efficiently neutralise the virus in MNT but are not detected by our indirect IgG MIAs.

In conclusion, when used in a multiplex MIA format, rEDIIIs were able to specifically identify WNV and TBEV antibodies, particularly if sera were sampled after D20 PI. EDIIIs of each virus were shown to be specific to the targeted virus, with no cross-reactivity observed between rEDIII beads during TBEV infection and slight cross-reactivity between JEV.EDIIII and WNV.EDIII beads on D58 with reference sera (Figure 4).

### 3.4. Evaluation of the MIA on Field Sera

*WNV Survey*. The study was based on sera sampled in countries (Pakistan and Madagascar) with a high prevalence of WNV infection.

Out of 101 sera positive with the ID Screen West Nile competition kit, 100 were found WNV.sE positive and one was WNV.sE negative with the flavivirus MIA. As regards binding of rEDIII beads on WNV.sE positive sera, 99 sera were found to bind WNV.EDIII, 53 JEV.EDIII, and 2 TBEV.EDIII. Concurrently, 99 sera neutralised WNV and 36 neutralised JEV during the MNT for WNV and JEV, respectively (Table 4). Cross-reactions were evidenced with both techniques and incited us to define rules for flavivirus differentiation.

By comparing the MFI of rEDIII beads and applying the rules defined in Section 3.2 (S/P ratio of WNV.EDIII at least 1.5-fold greater than S/P ratios of other rEDIII beads to be able to conclude that a WNV infection occurred), 96 sera were found WNV positive by MIA and four were undetermined. These results were similar to MNT results. The three negative MIA and MNT sera were low positive or doubtful in ELISA (% S/N = 38, 41, and 44, resp.).

To evaluate the specificity of the MIA on WNV negative samples, 104 sera sampled in 2007 in the Camargue region, Southern France, were added to the analysis [52]. This region is where French WNV cases had been previously reported in 2000 and 2004 but the tested sera were found negative by competition ELISA. One hundred and two sera were determined to be negative by the flavivirus MIA and two were found positive: one assigned as WNV positive and the other one positive for an undetermined flavivirus. These two discrepant results could be due to the generation of false positives by MIA or to MIA being more sensitive than ELISA. Interestingly, 56 other negative ELISA samples from Ireland—a country having never encountered WNV cases—also tested by MIA were all negative, supporting the second hypothesis.

In the case of our WNV serosurvey, the MIA offered sensitivity and specificity close to the ID Screen West Nile competition ELISA (Se = 99.0% and Sp = 98.1%) for the detection of flavivirus antibodies and similar to WNV MNT for the identification of WNV antibodies (Se = 100%) (Table 5).

*JEV Positive and Negative Sera from Japan*. Of the one hundred and one sera collected from Japan healthy horses vaccinated against JEV, the sensitivities of flavivirus ELISA and MIA were comparable: 90 sera were found WNV.sE positive by MIA, while they were either positive (88) or doubtful (2) by ELISA, and 11 sera were found negative by both MIA and ELISA. Fewer JEV positive sera than sera from the WNV survey reacted with WNV.EDIII (29 reactors) and TBEV.EDIII (2 reactors) and cross-reactivity in MNT was also evidenced in 11 sera, found positive for WNV and JEV antibodies.

The JEV MNT was shown to be more sensitive than MIA, with 91 sera found positive by JEV MNT versus 82 by MIA (Tables 4 and 5). This result is in agreement with MIA's lower sensitivity value (93.2%) calculated by ROC analysis and highlighted with reference sera.

JEV.sE (Genbank FJ979830.1) and WNV.sE (AY033389.1) proteins have a percentage of amino acid homology of 77% (calculated by NCBI, Blastp program). With our decision scheme, a sample was considered flavivirus positive only if WNV.sE bound equine sera. To improve the MIA, it could be beneficial to add beads coupled to other flaviviral antigens and in particular to JEV.sE and to determine its cut-off through comparison with a sensitive IgG ELISA dedicated to the detection of Japanese encephalitis disease. The addition of JEV.EDIII beads of JEV genotypes other than genotype 3 could also be considered to improve the technique's sensitivity.

Interestingly, three samples which were WNV.EDIII positive by MIA were found positive for both JEV and WNV by MNT. These three samples presented higher WNV neutralising antibody titres than JEV titres, but the comparison of end-point titres and the fourfold difference rule was only applicable for one sample considered as WNV positive. The other two sera gave inconclusive results following MNTs. It turned out that these three samples originated from horses born in the USA, while the remaining 98 horses were born either in Japan (96), in France (1), or in Ireland (1) and consequently these three horses could present sustained anti-WNV antibodies response, testimony of past vaccination, or exposure to WNV in the USA.

Finally, flavivirus MIA and ELISA presented a similar sensitivity for the detection of flavivirus antibodies in JEV positive sera, while their sensitivity was weaker than JEV MNT. For sera found to be positive for flavivirus (WNV.sE) by MIA, the identification of JEV positive sera by MIA and a JEV MNT were consistent. The determination of MIA diagnostic Se and Sp should now be validated through a large screening study with horses living in countries with a high JEV prevalence. 

*Austrian TBEV Survey*. Seventy-four sera collected in 2011 from horses in Austria and screened using flavivirus ELISA and WNV, USUV, and TBEV VNTs were tested with our new MIA technology [41].

Of 74 sera considered for analysis, low cross-reactivity between the rEDIIIs was observed, with seven sera reacting with the JEV.EDIII bead and one serum with the WNV.EDIII bead.

When considering TBEV.EDIII beads only, 61 sera were found to be positive and 13 negative, while ELISA identified 59 positive and three doubtful results and TBEV MNTs found 62 positive results (Table 4). All negative MIA samples were also found negative by ELISA and TBEV MNT (Sp = 100%).

Three samples found doubtful by ELISA and positive using a TBEV PRNT were found negative (1 sample), TBEV positive (1 sample), or with an undetermined flavivirus status by MIA (1 sample). The undetermined flavivirus status in this last sample was due to TBEV.EDIII and WNV.EDIII reacting beads (Figure 3). However this sample tested negative for WNV by MNT.

With these 74 samples, MIA was shown to be as sensitive as ELISA (Se = 100%) and slightly less sensitive than TBEV VNT (Se = 98.4%) on TBEV positive horse sera (Table 5).

## 4. Conclusion

In the present study, we describe an innovative multiplexing immunoassay (MIA) based on the use of recombinant antigens from zoonotic flaviviruses potentially responsible for neurological diseases in horses. The flaviviral antigens were efficiently produced in S2* Drosophila* cells and the resulting purified milligrams of recombinant WNV.sE or flavivirus rEDIII proteins were used for reproducible binding to coloured microspheres. The beads covered with viral antigens led to the specific capture of antibodies directed against WNV, JEV, or TBEV in spite of the well-known antigenic cross-reactivity between these flaviviruses. The flavivirus MIA provided a rapid and reliable alternative to routine methods for flavivirus serological diagnosis. As previously mentioned, standardised diagnostic tests such as WNV ELISAs or IFAs suffer from a lack of specificity, so positive results should be further investigated using time-consuming VNTs which require BSL3 facilities. Within three hours, our MIA method identified IgG directed against neurotropic flaviviruses with a sensitivity and specificity equivalent to an ELISA followed by corresponding VNTs. For JEV, a lack of sensitivity was underlined in our study, but could probably be resolved by the addition of JEV antigens (JEV.sE or rEDIII from other JEV genotypes).

The identification of the main neutralising epitopes involved in neutralisation of TBEV, WNV, and JEV in EDIII stimulated its use as an antigen for serological diagnosis of flavivirus infections [53]. The use of flavivirus rEDIII proteins in ELISA as a tool to detect and differentiate flavivirus infections already proved successful for tick- and mosquito-borne flaviviruses (Langat virus and WNV) as well as for mosquito-borne flaviviruses belonging to different serocomplexes (WNV and Wesselsbron virus) [54]. Indirect ELISA with WNV.EDIII was also shown to generate WNV-specific reactions with experimental sera from animals infected with flaviviruses of the same serocomplex (WNV, JEV, Saint Louis encephalitis, and Murray Valley encephalitis viruses) [20, 53]. However, in our hands, the OD signals produced by rEDIIIs ELISAs are not sufficiently dissimilar to differentiate the infecting flaviviruses when they belong to the same serocomplex, such as WNV and JEV. Moreover, the results of these rEDIIIs ELISAs usually need to be confirmed by a VNT to obtain accurate results [54]. By employing the multiplexing capacities of MIA, reactions in parallel and identical conditions could be easily compared and interpreted. In our study, MIA was shown to accurately identify TBEV, WNV, and JEV infections.

Several advantages plead in favour of the development and implementation of MIA technology in reference laboratories: not only does it remove the need for level 3 biocontainment conditions to confirm flavivirus infections, but it only requires a small quantity of sample (1 *μ*L) and is very quick (<3 hours compared to 3–6 days for flavivirus VNTs).

In the future, other proteins could be added to the flavivirus MIA protocol to improve the serological diagnosis of flaviviruses as coupling nonstructural proteins (NS) such as NS1 or NS5 to differentiate vaccinated from naturally infected animals or to distinguish recent from old infections [55]. The detection of antiflavivirus IgM as described in [36] is another promising approach. Finally, the development of species-independent MIAs for flaviviruses generally circulating in many different animal species would also be a valuable input.

## Figures and Tables

**Table 1:** Amount of flavivirus antigens produced in *Drosophila* S2 cells per litre of culture.

Recombinant flavivirus antigens	Amount of purified protein (mg) obtained per L of culture
WNV.sE	40
WNV.EDIII	175
JEV.EDIII	225
TBEV.EDIII	180

**Table 2:** Determination of MIA cut-off, diagnostic sensitivity, and diagnostic specificity for WNV.sE, WNV.EDIII, JEV.EDIII, and TBEV.EDIII antigens by ROC analysis on ELISA positive and negative samples.

	ROC analysis				
	Number of ELISA positive samples	Number of ELISA negative samples	ROC cut-off	Diagnostic sensitivity determined with this cut-off	Diagnostic specificity determined with this cut-off
WNV.sE	189	172	17	98.4	99.4
WNV.EDIII	101	172	54	97.0	92.3
JEV.EDIII	88	172	55	93.2	94.7
TBEV.EDIII	59	172	61	98.4	100.0

**Table 3:** Comparison of the earliest antibody detection using flavivirus ELISA, MIA, and VNT. Identification of specific WNV, JEV, or TBEV antibodies in reference sera sampled on days 0, 4, 8, 11, 14, 20, 35, and 58.

		ELISA positive^(1)^	MIA		MNT	
			Positive for WNV.sE^(2)^	Flavivirus identification^(3)^	Positive^(4)^	Flavivirus identification^(5)^
Sera	WNV lineage 1	D11	D11	D35	D8	D14
	WNV lineage 2	D11	D8	D8	D8	D14
	JEV TBEV	D20 D20	D20 D20	Not achieved D35	D20 D8	D35 D8

^(1)^ID Screen West Nile competition ELISA kit (ID Vet). Sample positive if %S/N ≤ 40%, doubtful if 40 ≤ %S/N < 50%, and negative if %S/N ≥ 50.

^(2)^WNV.sE positive if S/P ratio > 17 for the WNV.sE bead.

^(3)^WNV.EDIII identification if S/P ratio > 17 for the WNV.sE bead and S/P ratio > 54 for the WNV.EDIII bead.

JEV.EDIII identification if S/P ratio > 17 for the WNV.sE bead and S/P ratio > 55 for the JEV.EDIII bead; TBEV.EDIII identification if S/P ratio > 61 for the TBEV.EDIII bead.

^(4)^MNT positive if MNT titre ≥ threshold (10 for JEV and WNV and 20 for TBEV).

^(5)^MNT identification of the flavivirus with the highest neutralisation capacity and at least a fourfold difference in titres.

**Table 4:** Comparison of the identification of circulating flaviviruses in equine field sera by three different techniques: flavivirus ELISA, MIA, and VNT.

Positive sera	ELISA				MIA					Flavivirus identification		VNT						
	Number of field sera	ELISA pos.	ELISA doubt.	ELISA neg.	WNV.sE pos.	WNV.EDIII pos.	JEV.EDIII pos.	TBEV.EDIII pos.	Neg.			WNV pos.	JEV pos	USUV pos.	TBEV pos.	Neg.	Flavivirus identification	
WNV	103: Pak. (68) + Mada. (35)	101	*2 *	***0***	100	99	53	2	***3***	96	WNV positive	99	36	9/35 (Mada.)	NA^*∗*^	***4***	96	WNV positive
										4	Undetermined flavivirus						3	Undetermined flavivirus
JEV	101 (Japan)	88	*2 *	***11***	90	29	87	2	***11***	82	JEV positive	11	96	NA	NA	***5***	91	JEV positive
										3	WNV positive						1	WNV positive
										5	Undetermined flavivirus						4	Undetermined Flavivirus
TBEV	74 (Austria)	59	*3 *	***12***	59	1	7	61	***13***	60	TBEV positive	3	NA	4	62	***12***	62	TBEV positive
										1	Undetermined flavivirus							

Negative sera	160 (Camargue + Ireland)			***160***	2	16^*∗∗*^	12^*∗∗*^	0	***158***	***158***	Negative							
										2	WNV positive							

^*∗*^Not analysed (NA).

^*∗∗*^Positive sera evaluated as false reactives for JEV or WNV if WNV.sE signal was negative.

Bold italic: negative sample and italic: doubtful sample.

The thresholds for ELISA, MIA, and MNT were as defined in Table 3. In the event of cross-reaction with rEDIII beads during MIA, a horse was considered infected with a specific flavivirus if the corresponding bead coupled to rEDIII generated an S/P ratio at least 1.5-fold greater than that generated with the other rEDIII beads. In the event of cross-reaction with MNT, flavivirus identification is determined by the virus with the highest neutralisation capacity and at least a fourfold difference in titres.

**Table 5:** Comparison of Se and Sp for flavivirus MIA, ELISA, and VNT on horse field sera.

Virus identified	MIA/ELISA	MIA/VNT		Number of field sera tested
	Positive for WNV.sE/ELISA positive^(*∗*)^	Positive for WNV.sE/VNT positive^(*∗*)^	MIA identification/ VNT identification^(*∗*)^	

WNV	Se = 99.0% (100/101) Sp = 98.1% (102/104)^(1)^	Se = 100.0% (99/99)	Se = 100.0% (96/96)	207 sera from Pakistan (68) + Madagascar (35) + France (104)
JEV	Se = 100.0% (88/88)^(2)^ Sp = 100.0% (11/11)	Se = 93.8% (90/96) Sp = 80.0% (4/5)^(3)^	Se = 90.1% (82/91)^(4)^	101 sera from Japan

	Positive for WNV.sE/ELISA positive^(*∗*)^	Positive for TBEV.EDIII/VNT positive^(*∗∗*)^	MIA identification/ VNT identification^(*∗*)^	

TBEV	Se = 100.0% (59/59)^(5)^ Sp = 100.0% (12/12)	Se = 98.4% (61/62) Sp = 100.0% (12/12)	Se = 96.8% (60/62)^(6)^ Sp = 100.0% (12/12)	74 sera from Austria

^(*∗*)^Same thresholds as in Table 3.

^(*∗∗*)^Positive for TBEV when the TBEV.EDIII MFI > 61.

^(1)^Two samples from the Camargue, France, were found positive by MIA but negative by ELISA.

^(2)^Two samples found doubtful by ELISA and positive by MIA were not taken into account for Se calculation (ELISA/MIA).

^(3)^One sample was found doubtful by ELISA, slightly positive by MIA with the WNV.sE bead, and negative by MNT.

^(4)^Three samples were identified as positive for WNV by MIA. One was also positive for WN by MNT while the other two were positive for an undetermined flavivirus.

^(5)^Three samples found doubtful by ELISA and positive (2) or negative (1) by MIA were not taken into account for Se calculation (ELISA/MIA).

^(6)^One undetermined sample by MIA due to TBEV.EDIII and WNV.EDIII reacting beads.
